# The Herald Bleed—A Fortunate Endoscopic Diagnosis of an Aorto‐Duodenal Fistula Secondary to a Mycotic Aortic Aneurysm With Abscess Formation

**DOI:** 10.1002/ccr3.70615

**Published:** 2025-07-08

**Authors:** Arteen Arzivian, Robert O'Neill, Ian Lockart

**Affiliations:** ^1^ Gastroenterology and Hepatology Department St. Vincent's Hospital Sydney New South Wales Australia; ^2^ Faculty of Medicine, Health and Human Sciences, Macquarie Medical School Macquarie University Sydney New South Wales Australia; ^3^ Faculty of Medicine, St. Vincent's Clinical School University of new South Wales Sydney New South Wales Australia

**Keywords:** aorta, duodenum, fistula, hemorrhage, upper gastrointestinal bleeding

## Abstract

Aorto‐duodenal fistula is a rare but catastrophic cause of gastrointestinal bleeding. A high index of suspicion is required for diagnosis. Reviewing previous imaging provides essential hints. A pulsatile clot or bleeding source can be seen on endoscopy. Management is surgical with multi‐disciplinary involvement. Unfortunately, mortality remains high.

## 1 Introduction

Upper gastrointestinal bleeding (UGIB) is a potentially life‐threatening medical emergency that usually requires an endoscopic assessment and treatment. The most common causes of UGIB remain peptic ulcer disease, variceal bleeding, Mallory‐Weiss tear, oesophagitis, duodenitis, and vascular lesions [1]. The more challenging presentation includes uncommon causes of UGIB, which represent 5%–10% of all cases; these include Dieulafoy's lesion, homosuccus pancreaticus, gastrointestinal amyloidosis, and other disorders [2]. In this case report, we present a patient with catastrophic UGIB secondary to an aorto‐enteric fistula that was suspected, recognized, and treated promptly, leading to a successful outcome for such a rare disease that is usually associated with a poor prognosis, mainly due to a delayed diagnosis.

## 2 Case History/Examination

A 77‐year‐old male developed acute haematemesis and haematochezia with associated haemodynamic instability. This was in the context of a recent protracted admission with *Streptococcus pneumoniae* discitis and bacteraemia managed with intravenous antibiotics.

## 3 Methods (Investigations and Treatment)

**FIGURE 1:**
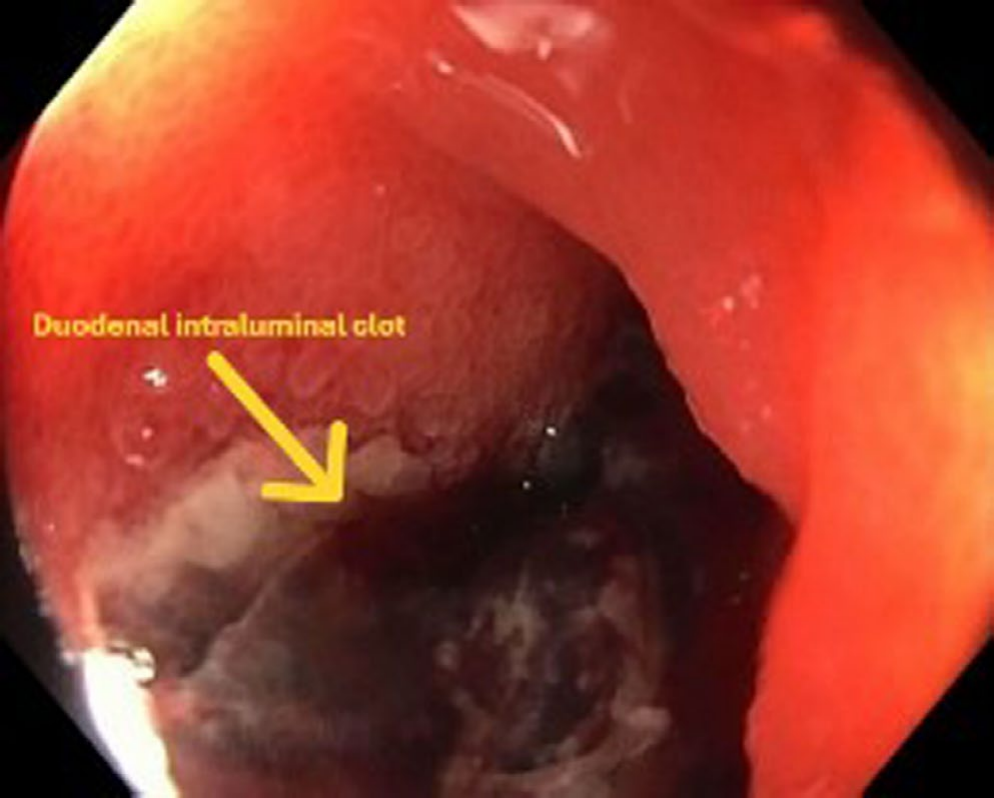
Upper gastrointestinal endoscopy image showing adherent clot in the third part of the duodenum.

**FIGURE 2:**
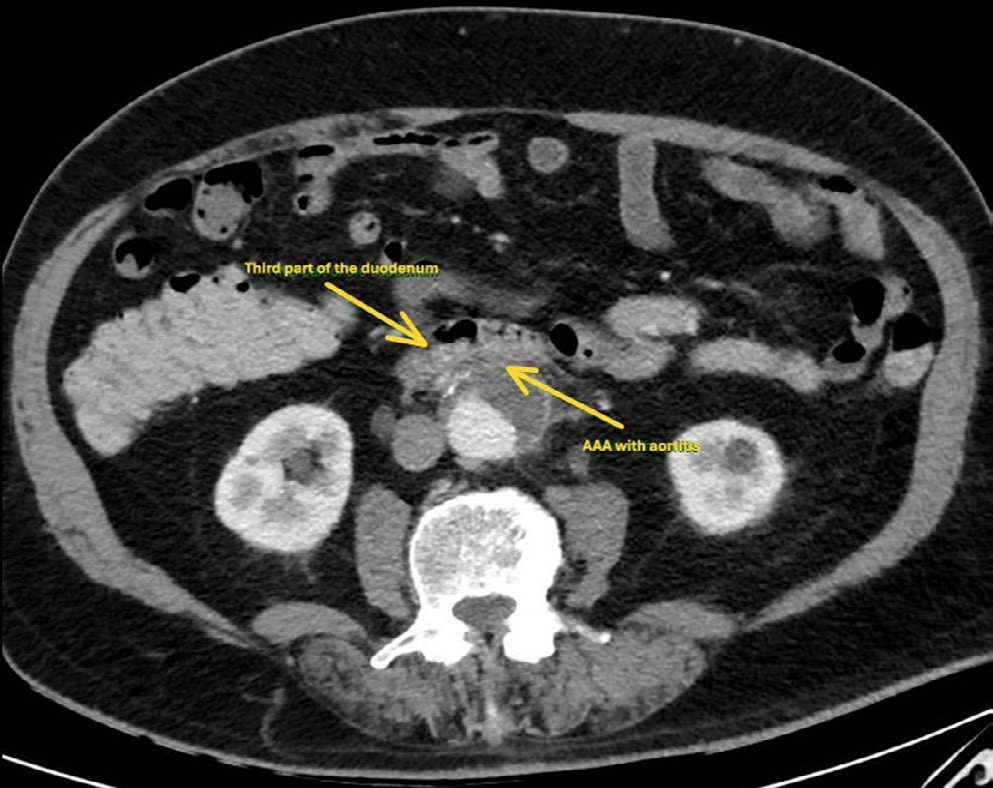
A computed tomography image of an infra‐renal saccular abdominal aortic aneurysm with features of aortitis in the aortic wall opposing the duodenum.

**FIGURE 3:**
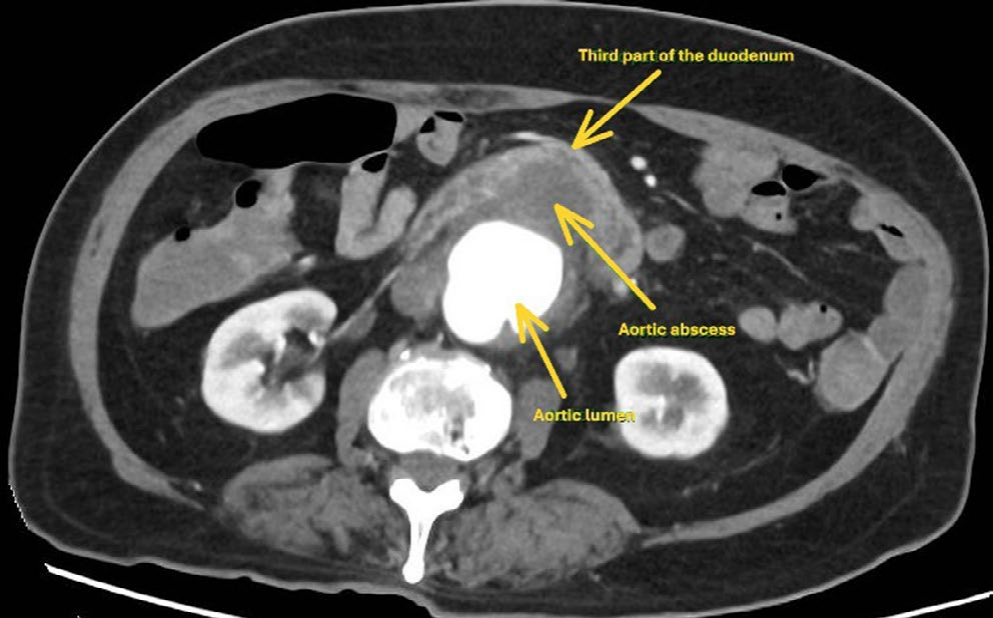
A computed tomography image showing the development of aortic abscess between the abdominal aortic aneurysm and the third part of duodenum.

After appropriate resuscitation, an urgent gastroscopy was arranged, which demonstrated frank blood in the stomach and proximal duodenum without an obvious source of bleeding. Further assessment of the duodenum demonstrated luminal stenosis at the second portion of the duodenum. Passage of the endoscope to the third portion of the duodenum was challenging due to luminal stenosis; however, it was subsequently achieved with push enteroscopy. A large pulsatile adherent clot in the third part of the duodenum was identified without active bleeding (Figure 1). An abdominal computed tomography (CT) scan from the previous admission was reviewed peri procedurally, which demonstrated an infra‐renal saccular abdominal aortic aneurysm (AAA) with features of aortitis in the wall opposing the duodenum (Figure 2). An urgent CT Aortogram was arranged, which revealed an aortic abscess between the AAA and the third part of the duodenum (Figure 3). The patient was taken for urgent operative repair, with intra‐operative exploration revealing a 1 cm defect in the posterior duodenal wall, confirming the diagnosis of an aorto‐duodenal fistula secondary to a mycotic AAA. Duodenal wall repair and aortic grafting were undertaken successfully.

## 4 Conclusions and Results (Outcome)

The patient's postoperative period was uncomplicated, with no further episodes of gastrointestinal bleeding. He spent a few days in the intensive care unit for monitoring and then was discharged to the medical ward, followed by a transfer for further rehabilitation before returning to his usual residence.

## 5 Discussion

Aorto‐enteric fistula was first described by Sir Astley Cooper in 1826 when he defined it as a connection between the two organs by the aorta eroding through the duodenum [3]. It can be either a primary spontaneous aorta‐enteric fistula, similar to our case report, or secondary, related to complications of previous aortic grafts [4]. It is difficult to determine the exact incidence of this condition as most of the data available are case reports. It usually presents with a first sentinel hemorrhage followed by a second catastrophic bleeding, which can be fatal. Upper gastrointestinal endoscopy is reported to have a low detection rate. Computed tomography is considered the most widely used imaging modality for investigating the presence of a fistula; however, the exact sensitivity and specificity are reported variably and have not been confirmed in studies. Endoscopic interventions have no role in the treatment of aorto‐enteric fistulas. Surgical interventions were the primary modality of management in patients with active bleeding. Angiography and endovascular interventions revolutionized the treatment of this condition. Despite the new interventions and improvements in medical technologies, the mortality rate remains high [5]. A retrospective review of patients with aorto‐enteric fistula over 20 years in the United States of America reported a 30‐day mortality of 35% and 1 and 3‐year survival of approximately 50% despite surgical interventions [6].

A case series of nine patients from China reported predominance in males above the age of 60, and they were investigated and managed similarly to our patient; however, in contrast to our patient, the rate of perioperative complications was high, which was avoided in our case due to a high index of suspicion, prompt, and efficient management [7].

## Author Contributions

**Arteen Arzivian:** conceptualization, data curation, project administration, software, visualization, writing – original draft, writing – review and editing. **Robert O'Neill:** conceptualization, investigation, visualization, writing – review and editing. **Ian Lockart:** conceptualization, project administration, supervision, writing – review and editing.

## Ethics Statement

The authors are accountable for all aspects of the work and ensure that questions related to the accuracy or integrity of any part of the work are appropriately investigated and resolved. This study was exempted from ethics approval by the St Vincent's Hospital Human Research Ethics Committee as it is a case report.

## Consent

A written informed consent was obtained directly from the patient.

## Conflicts of Interest

The authors declare no conflicts of interest.

## Data Availability

The data supporting this study's findings are available upon reasonable request from the corresponding author. The data are not publicly available due to privacy and ethical restrictions.
